# Copper, Ceruloplasmin, Zinc, and Manganese Levels in Brain and Biological Fluids from Parkinson’s Disease Patients: Systematic Review and Meta-Analysis

**DOI:** 10.3390/cells15030288

**Published:** 2026-02-03

**Authors:** Félix Javier Jiménez-Jiménez, Hortensia Alonso-Navarro, Elena García-Martín, Miguel Angel Martín-Gómez, Paula Salgado-Cámara, Alba Cárcamo-Fonfría, Margarita Arroyo-Solera, José A. G. Agúndez

**Affiliations:** 1 Section of Neurology, Hospital Universitario del Sureste, Ronda del Sur 10, 28500 Arganda del Rey, Madrid, Spain; hortalon@yahoo.es (H.A.-N.); mmartingomez7@salud.madrid.org (M.A.M.-G.); paula.salgado@salud.madrid.org (P.S.-C.); alba.carcamo@salud.madrid.org (A.C.-F.); margarita.arroyo@salud.madrid.org (M.A.-S.); 2 University Institute of Molecular Pathology Biomarkers, Universidad de Extremadura, 10071 Cáceres, Spain; elenag@unex.es

**Keywords:** Parkinson disease, copper, ceruloplasmin, zinc, manganese, brain, cerebrospinal fluid, serum/plasma, urine, hair

## Abstract

The present systematic review and meta-analysis aims to establish whether the brain, cerebrospinal fluid (CSF), serum/plasma whole blood, urine, and hair levels of copper, ceruloplasmin, zinc, and manganese are related to the risk for Parkinson’s disease (PD). We reviewed the PubMed and Web of Science Core Collection databases from 1966 to 29 November 2025, and identified references of interest for this topic. We performed the meta-analysis of eligible studies that followed the PRISMA and MOOSE guidelines, with the R software package meta R 4.2.0 version. When compared to age- and sex-matched controls, PD patients showed decreased concentrations of copper in the *substantia nigra* and other brain areas, a trend towards increased CSF and decreased serum/plasma copper levels, decreased serum/plasma ceruloplasmin levels, decreased zinc levels in serum/plasma and increased zinc in whole blood and hair, and increased hair manganese levels. These results suggest an association between these transition metals and risk for PD.

## 1. Introduction

The main neuropathological features of Parkinson’s disease (PD)—the neurodegeneration of the dopaminergic neurons at the substantia nigra compacta—and the presence of Lewy bodies (composed of alpha-synuclein aggregates), are well established. However, its etiology and the pathogenetic mechanisms leading to the neurodegenerative processes are not well-known. The interplay of genetic and environmental factors could hypothetically induce a dysregulation of multiple cellular pathways, including oxidative stress, mitochondrial dysfunction, neuroinflammation, trophic factor deficiency, excitotoxicity, alterations in the mechanisms implicated in protein clearance, intracellular calcium accumulation, synaptic dysfunction, etc., that would eventually result in neuronal and glial cell death [1,2]. Many reports published in the last decade have suggested that oxidative stress (defined as the imbalance between the production of free radicals and diverse mechanisms against oxidative processes)—with the involvement of transition metals such as copper, zinc, manganese, and iron—plays an important role in the pathogenesis of PD [3,4,5,6].

A meta-analysis of brain copper and zinc concentrations in post-mortem tissue showed decreased copper and similar zinc levels in the substantia nigra of PD patients compared to controls [7]. Another meta-analysis, including only works published between 2011 and 2022, reported “lower copper content in postmortem brain tissue”, by mixing the copper concentrations found in different tissues from different studies [8]. However, findings from meta-analyses assessing copper, zinc, and manganese concentrations in cerebrospinal fluid (CSF), serum, plasma, and urine of patients with PD compared to controls have been inconsistent. Two studies reported no significant differences in CSF, serum, or plasma levels of zinc, copper, and manganese [3,7]. Similarly, Adani et al. [9] found no significant differences in CSF and serum/plasma copper levels, as well as in CSF zinc levels. In contrast, other authors described an association between reduced serum/plasma zinc concentrations and increased PD risk [9,10,11]. They also reported a trend toward lower serum copper concentrations in PD patients compared to controls [8].

In the current meta-analysis, we analyzed the results of studies on the concentrations of copper, ceruloplasmin, zinc, and manganese in different brain regions, in the CSF, in serum/plasma, urine, and hair of PD patients compared to controls. For this purpose, we analyzed the pooled results of studies giving data on the absolute concentrations of these variables.

## 2. Methods

### 2.1. Search Strategy

We performed a complete literature search, without any language restrictions, using two Databases (PubMed and the Core Collection of Web of Science) up to 29 November 2025. The search strategy used the term “Parkinson’s Disease” that was crossed with combinations of the terms “brain”, “cerebrospinal fluid”, “serum”, “plasma”, “blood”, “urine”, “hair”, “copper”, “ceruloplasmin”, “zinc”, and “manganese”. Table 1 summarizes the number of references obtained for each combination. The whole search retrieved a total of 2023 items.

### 2.2. Criteria for Eligibility and Exclusion of Studies from the Meta-Analyses

We screened the abstracts of all original articles; publications in abstract form were excluded from further analysis. The initial selection included 85 studies measuring brain, CSF, serum/plasma, urine, and/or hair concentrations of copper, ceruloplasmin, zinc, or manganese [12,13,14,15,16,17,18,19,20,21,22,23,24,25,26,27,28,29,30,31,32,33,34,35,36,37,38,39,40,41,42,43,44,45,46,47,48,49,50,51,52,53,54,55,56,57,58,59,60,61,62,63,64,65,66,67,68,69,70,71,72,73,74,75,76,77,78,79,80,81,82,83,84,85,86,87,88,89,90,91,92,93,94,95,96,97,98]. The authors, year of publication, methods used to perform the determinations, and observations (including the reasons for exclusion from the meta-analysis) when appropriate, were summarized in Table 2.

**Figure 1:**
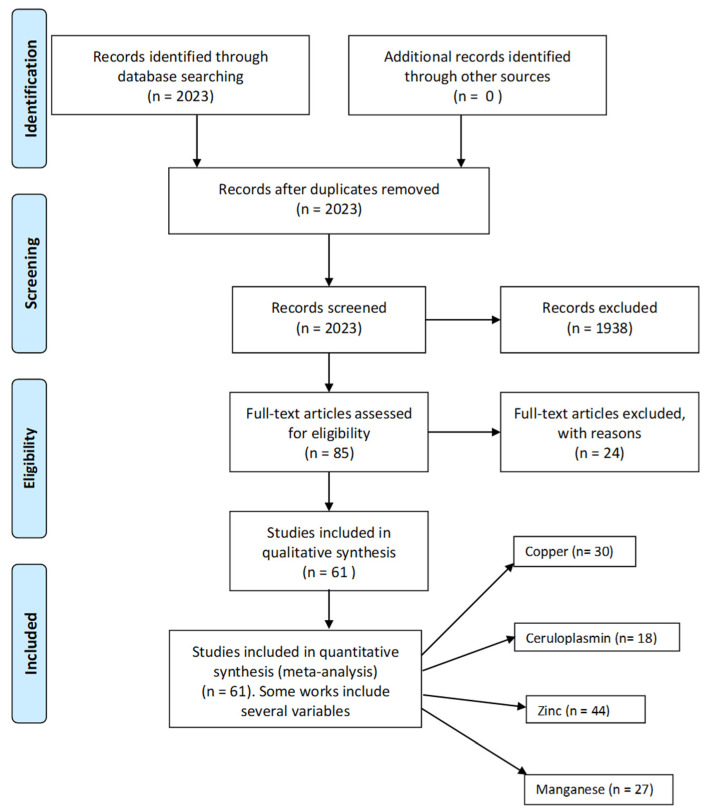
Flowchart for selection of studies.

Some articles were excluded, totally or partially, from the meta-analyses because of the following reasons: (A) total or partial overlapping with other articles from the same group [31,51,52,53,75,78]; (B) lack of a control group [55,64,74,75,88,94]; (C) lack of part of the data in the text, tables or figures [21,58,60,89,91,92,96]; (D) measurements given in units different from those used in most articles without the possibility of conversion [16,32,49,80], and (E) inability to obtain the original article [22]. The flowchart for the selection of the studies is plotted in Figure 1.

Selected case–control studies reporting brain, CSF, serum/plasma, urine, and hair concentrations of copper, ceruloplasmin, zinc, and manganese are summarized in Supplementary Table S1. The results of all studies were given as mean ± SD values. All concentrations were standardized to the same units across studies, converting values when necessary. The odds ratios (OR) and 95% confidence intervals (95% CI), and the statistical significance of each individual study, were calculated.

### 2.3. Statistical Analysis

Meta-analyses were carried out using the R software package meta R 4.2.0 version. [99] and followed by the PRISMA [100] (Supplementary Table S2) and MOOSE guidelines [101] (Supplementary Table S3), to maintain methodological transparency, accuracy, and reproducibility. The review protocol was not registered in PROSPERO due to the rapid timeline of the project and because the scope of the study, which involved synthesizing previously published datasets, did not involve patient-level intervention or outcomes requiring prospective pre-registration. Because of the observed high heterogeneity across studies, we used the random-effects model to calculate the *p* values, although the plots also included the common effects models for comparison. The meta-analytical procedure used was the inverse variance method, with DerSimonian-Laird estimator for Tau^2^, Jackson method for the confidence interval of Tau^2^ and Tau, and Hedges’ g (bias-corrected standardized mean difference). We calculated the statistical power to detect differences in mean values (alpha = 0.05) for the pooled samples when stated in the text.

## 3. Results

### 3.1. Copper

#### 3.1.1. Brain

**Figure 2:**
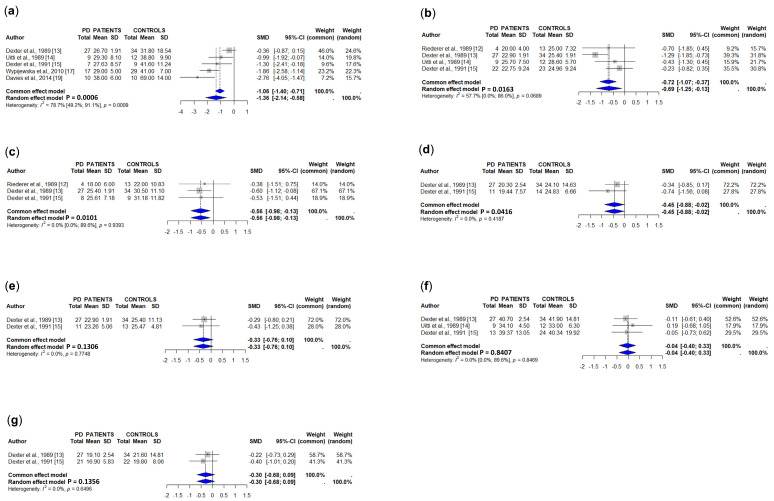
Meta-analyses of studies on concentrations of copper [12,13,14,15,17,19] in the *substantia nigra* (**a**), caudate nuclei (**b**), putamen (total) (**c**), *globus pallidus medialis* (**d**), *globus pallidus medialis* (**e**), cerebellum (**f**), and cerebral cortex (**g**) in PD patients and controls.

The results of studies assessing copper concentrations in different brain regions are summarized in Table 2 and Supplementary Table S1. In the meta-analysis, we only included those studies in which copper concentrations were expressed in μg/g tissue or could be converted to this unit. A total of five studies measuring copper levels in the *substantia nigra* (total), involving 70 PD patients and 94 controls [13,14,15,17,19], showed a significant decrease in PD patients (Figure 2a). Loeffler et al. [16] also reported decreased copper concentrations in the substantia nigra of PD patients expressed as ng/μg of protein [16], and Genoud et al. [21] reported a 54% decrease in copper in the substantia nigra of PD patients, but full data were not available. In contrast, another two studies showed similar copper concentrations between PD patients and controls assessed by ceruloplasmin staining [80] and synchrotron radiation-based X-ray fluorescence [18]. Dexter et al. [13] reported decreased copper levels in the *substantia nigra pars compacta* from PD patients, and Riederer et al. [12] did not find significant differences in the copper concentrations in the *pars oralis* or in the *pars caudalis* of the *substantia nigra* between PD patients and controls.

The pooled data of four studies, involving 62 PD patients and 82 controls [12,13,14,15], showed a significant decrease in copper concentrations in the caudate of PD patients (Figure 2b). Another study showed decreased caudate copper levels in PD patients expressed as ng/μg of protein [16], and another did not find significant differences between PD patients and controls measured as ceruloplasmin staining [80].

Copper concentrations in total putamen are decreased in PD patients compared to controls in pooled data from three studies [12,13,15] involving 39 PD patients and 59 controls (Figure 2c). In one of these studies, the concentrations of copper in medial and in lateral putamens did not, however, show significant differences between study groups [13]. Copper levels were also found to decrease in PD patients at *globus pallidus medialis* [13,15], *globus pallidus lateralis* [13,15], and cerebellum [13,14,15] (Figure 2d, Figure 2e and Figure 2f, respectively), whereas copper levels were similar in PD patients and controls in the cerebral cortex [13,15] (Figure 2g). Finally, some individual studies showed decreased copper concentrations in the raphe plus reticular formation [12], red nucleus [12], locus ceruleus [19], olfactory bulb [20], and amygdala [12], and increased copper levels in the CA1 area [80] (Table 2 and Supplementary Table S1).

#### 3.1.2. Cerebrospinal Fluid

**Figure 3:**
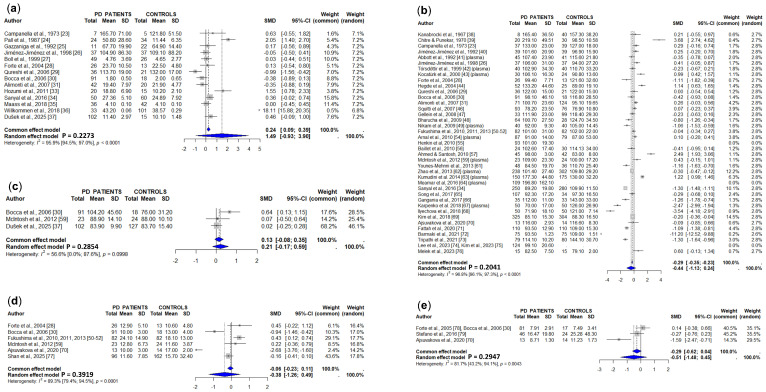
Meta-analysis of studies measuring CSF (**a**), serum/plasma (**b**), whole blood (**c**), urine (**d**), and hair (**e**) copper concentrations in PD patients and controls [23,24,25,26,27,28,29,30,31,33,34,35,36,37,38,39,40,41,42,43,44,46,47,48,49,50,51,52,54,55,56,57,59,61,62,63,64,65,66,67,68,69,70,71,72,73,74,75,76,77,78,79].

Sixteen studies addressed CSF copper levels, but two of them were excluded from the meta-analysis because of the previously mentioned reasons (Table 2 and Supplementary Table S1) [22,32]. One of them found CSF copper concentrations decreased [29], four were increased [24,33,36,37], and nine were similar in PD patients compared to controls [23,25,26,27,28,30,31,34,35]. The final meta-analysis, which involved 564 PD patients and 429 controls, showed a non-significant increase in PD patients according to the random effects model, although the common effect model indicates that the difference is significant (*p* = 0.0021) (Figure 3a).

#### 3.1.3. Serum/Plasma

Forty-two studies addressed serum (32 studies) or plasma (10 studies) copper concentrations (Table 2 and Supplementary Table S1); it was significantly increased in PD patients in seven studies [31,39,43,44,57,59,63], decreased in fourteen studies [28,30,45,48,49,61,62,66,67,68,69,71,72,73], and similar to those of controls in seventeen studies [23,26,29,34,38,40,41,42,46,47,50,51,52,54,59,70,76]. Four studies [55,64,74,75] showed results of PD patients but lacked control groups. The final meta-analysis involved 2894 PD patients and 2549 controls, and showed a slight, non-significant decrease in serum/plasma copper concentrations in PD patients compared to controls according to the random effects model, although the common effect model indicates that the difference is significant (*p* < 0.0001) (Figure 3b).

#### 3.1.4. Whole Blood

Bocca et al. [30] showed increased concentrations of copper in the whole blood of PD patients, while another two studies showed similar levels between PD patients and controls (Table 2 and Supplementary Table S1) [37,59]. The pooled data from these three studies (216 PD patients and 169 controls) showed a non-significant increase in copper concentrations in PD patients (Figure 3c).

#### 3.1.5. Urine

Copper concentrations were found to be increased in the urine in PD patients in one study [50,51,52], decreased in two [30,70], and similar to those of controls in another three studies (Table 2 and Supplementary Table S1) [28,59,77]. The pooled data of these studies, involving 331 PD patients and 313 controls, showed non-significant differences between PD patients and controls (Figure 3d).

#### 3.1.6. Hair

Ajsuvakova et al. [70] found decreased concentrations of copper in the hair of PD patients, while other authors did not find significant differences between PD patients and controls (Table 2 and Supplementary Table S1) [30,78,79]. The meta-analysis, which involved 140 PD patients and 55 controls, did not show significant differences (Figure 3e).

### 3.2. Ceruloplasmin

#### 3.2.1. Brain

Loeffler et al. [16] reported increased concentrations of copper in the caudate, putamen, substantia nigra, hippocampus, temporal, frontal, and parietal cortex, and cerebellum from PD patients compared to controls (Table 2 and Supplementary Table S1). Data from this study have not been replicated to date.

#### 3.2.2. Cerebrospinal Fluid

Only two studies with a small sample size analyzed CSF ceruloplasmin levels in PD and controls, one of them showing these values as increased in PD [81] and the other indicating non-significant differences [23] (Table 2 and Supplementary Table S1). The pooled data (31 PD patients and 14 controls) showed non-significant differences between PD patients and controls.

#### 3.2.3. Serum/Plasma

**Figure 4:**
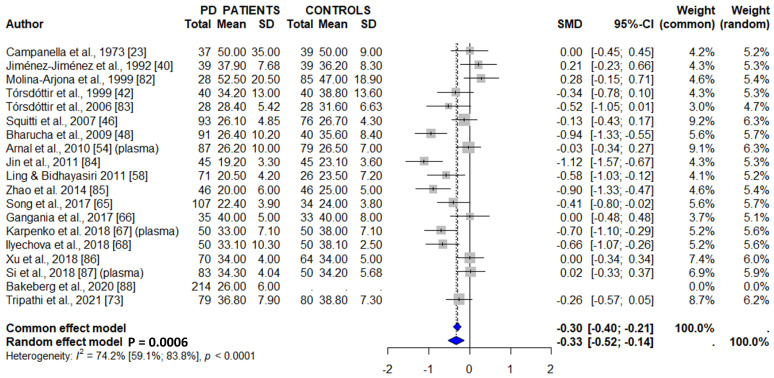
Meta-analysis of studies measuring serum or plasma ceruloplasmin levels in PD patients and controls [23,40,42,46,48,54,58,65,66,67,68,73,82,83,84,85,86,87,88].

Ceruloplasmin levels were assessed in serum by sixteen studies and in plasma by another three studies (Table 2 and Supplementary Table S1). Six studies showed decreased serum/plasma ceruloplasmin levels in PD patients [48,65,67,68,84,85], while another twelve did not show significant differences [33,40,42,46,54,58,66,73,82,83,86,87]. Another study [88] was not included in the meta-analysis because it lacked a control group. The pooled data (1079 PD patients and 904 controls) showed a significant decrease in serum/plasma ceruloplasmin levels in PD patients as compared to controls (Figure 4).

### 3.3. Zinc

#### 3.3.1. Brain

**Figure 5:**
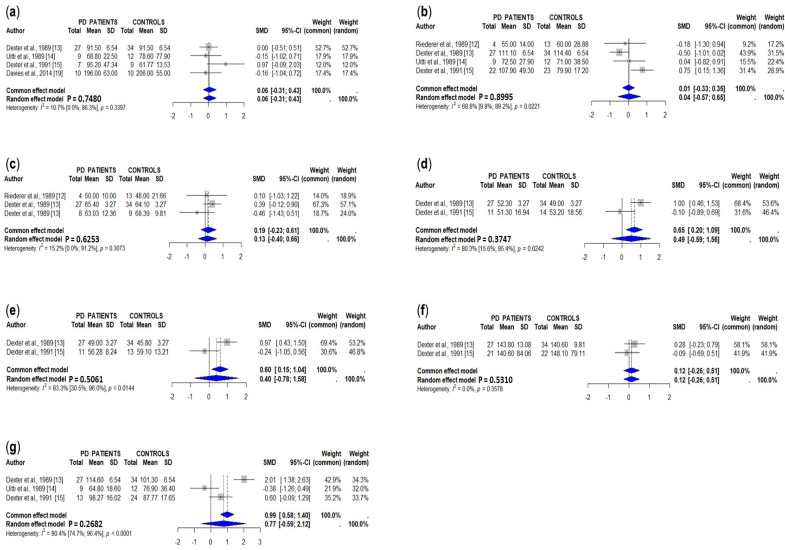
Meta-analyses of studies on concentrations of zinc [12,13,14,15,19] in the *substantia nigra* (**a**), caudate nuclei (**b**), putamen (total) (**c**), *globus pallidus medialis* (**d**), *globus pallidus medialis* (**e**), cerebellum (**f**), and cerebral cortex (**g**) in PD patients and controls.

Table 2 and Supplementary Table S1 summarize the results of studies assessing concentrations of zinc in different brain regions. The meta-analysis included only those studies in which zinc concentrations were expressed in μg/g tissue or could be converted to this unit. Four studies involving 53 PD patients and 65 controls [13,14,15,19] measuring zinc levels in the *substantia nigra* (total) showed non-significant differences between PD patients and controls (Figure 5a). Mann et al. [90] showed non-significant differences in zinc concentrations in the *substantia nigra* in the two study groups expressed as ng/μg of protein. Dexter et al. [13] reported similar zinc levels in the *substantia nigra pars compacta* from PD patients and controls, and, similarly, Riederer et al. [12] did not find significant differences in the copper concentrations in either the *pars oralis* or the *pars caudalis* of the *substantia nigra*.

Concentrations of zinc in caudate nuclei (Figure 5b) [12,13,14,15], putamen (Figure 5c) [12,13,15], and *globus pallidus medialis* (Figure 5d) [13,15] were similar in PD patients and controls. In the *globus pallidus lateralis*, there was a significant increase in PD patients with the use of the common effects model (*p* = 0.0088), but not with the random effects model (Figure 5e) [13,15]; in the cerebral cortex (Figure 5f) [13,15] they were similar in PD patients and controls. Cerebellum zinc levels were increased in PD patients, although this finding was not significant using the random effects model, the common effects models indicated a *p* value < 0.001 (Figure 5g) [13,14,15]. Some individual studies described non-significant differences in zinc levels between PD patients and controls in putamen medial [13], putamen lateral [13], globus pallidus total [12], frontal cortex [14], occipital cortex [19], amygdala [12], cingulate gyrus [12], raphe plus reticular formation [12], red nucleus [12], and ceruleus [19], olfactory bulb [20], and olfactory tract [20].

#### 3.3.2. Cerebrospinal Fluid

**Figure 6:**
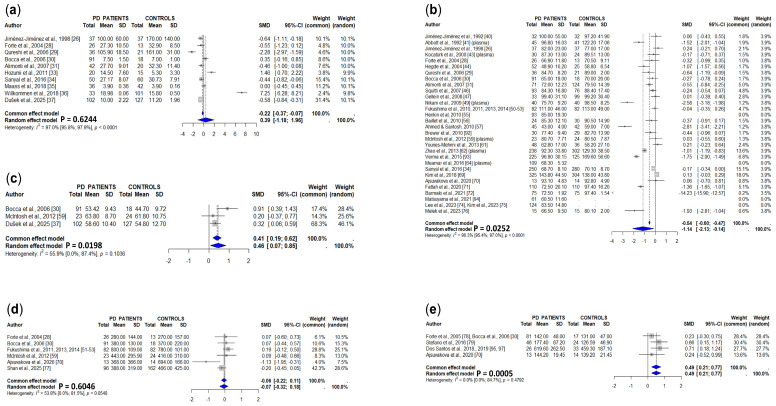
Meta-analysis of studies measuring CSF (**a**), serum/plasma (**b**), whole blood (**c**), urine (**d**), and hair (**e**) zinc concentrations in PD patients and controls [26,28,29,30,31,33,34,35,36,37,40,41,43,44,46,47,49,50,51,52,53,55,56,57,59,61,62,64,69,70,71,72,75,76,77,92,93,94,95,97].

CSF zinc levels were addressed in ten studies (Table 2 and Supplementary Table S1), being significantly decreased in PD patients in four of them [26,29,34,37], increased in two [33,36], and similar to those of controls in another four [28,30,31,35]. The pooled results, involving 473 PD patients and 454 controls, showed a non-significant trend towards increased CSF zinc concentrations in PD patients, although the differences were significant in the common effects model (*p* = 0.0034) (Figure 6a).

#### 3.3.3. Serum/Plasma

Thirty-four studies addressed serum (twenty-eight studies) or plasma (four studies) zinc concentrations (Table 2 and Supplementary Table S1). Eleven of them reported decreased zinc levels in PD patients [29,31,41,44,49,57,62,71,72,76,93], and eighteen did not find significant differences between PD patients and controls [26,28,30,34,40,43,46,47,50,51,52,53,56,59,61,69,70,92]. Four studies did not include a control group [64,74,75,94] and therefore were not included in the meta-analysis. The final meta-analysis, which included 2476 PD patients and 2018 controls, showed a significant decrease in serum/plasma zinc levels in PD patients (Figure 6b).

#### 3.3.4. Whole Blood

Two studies showed increased concentrations of zinc in whole blood from PD patients compared to controls [30,37], and another did not find significant differences (Table 2 and Supplementary Table S1) [59]. The pooled data showed a significant trend towards increased zinc concentrations in whole blood from PD (Figure 6c).

#### 3.3.5. Urine

Six studies addressed urine zinc levels. Only one of these studies showed a significant decrease in PD patients [70], and the others did not show significant differences between PD patients and controls (Table 2 and Supplementary Table S1) [20,28,51,52,53,59,77]. The results of the meta-analysis showed a non-significant decrease in PD patients (Figure 6d).

#### 3.3.6. Hair

The pooled data of four studies addressing hair zinc levels did show a significant increase in this value in PD patients (Table 2 and Supplementary Table S1, Figure 6e).

### 3.4. Manganese

#### 3.4.1. Brain

Two individual studies did not show significant differences between PD patients and controls in the concentration of manganese in several brain areas, including the frontal cortex [14], caudate [14], substantia nigra [14], cerebellum [14], olfactory bulb [20], and olfactory tract [20] (Table 2 and Supplementary Table S1).

#### 3.4.2. Cerebrospinal Fluid

**Figure 7:**
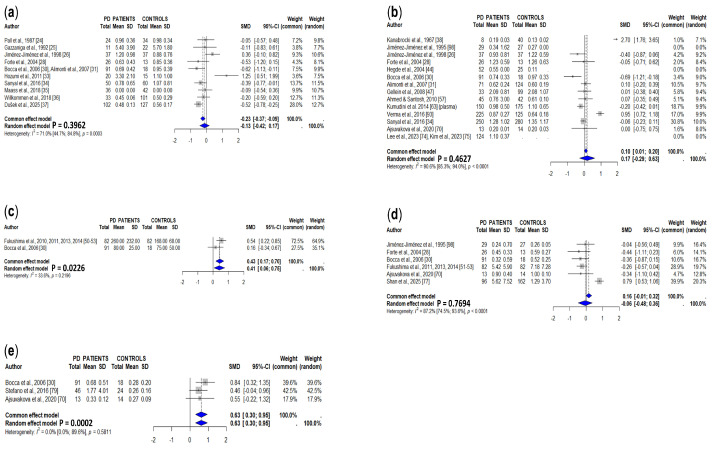
Meta-analysis of studies measuring CSF (**a**), serum/plasma (**b**), whole blood (**c**), urine (**d**), and hair (**e**) manganese concentrations in PD patients and controls [24,25,26,28,30,31,33,34,35,36,37,38,44,47,50,51,52,53,57,63,70,74,75,77,79,93,98].

CSF manganese levels were addressed by ten studies (Table 2 and Supplementary Table S1), four of them showing decreased CSF Mn in PD patients [30,31,32,33,34,37] and others showing non-significant differences when compared to controls [24,25,26,28,35,36,37]. The analysis of pooled data did not show significant differences between PD patients and controls in the random effects models, although it did in the common effects model (*p* = 0.0013) (Figure 7a).

#### 3.4.3. Serum/Plasma

Thirteen studies assessed serum and one analyzed the plasma levels of Mn in PD patients; two of the studies lacked a control group (Table 2 and Supplementary Table S1). The meta-analysis of eligible studies, including 1154 PD patients and 994 controls, showed a non-significant increase in serum/plasma manganese in PD patients, which was marginally significant in the common effects model (*p* = 0.0333) (Figure 7b).

#### 3.4.4. Whole Blood

Whole blood manganese levels were addressed by two groups [30,50,51,52,53] (Table 2 and Supplementary Table S1), which were higher in PD patients than in controls (Figure 7c).

#### 3.4.5. Urine

Urine manganese levels were researched in six studies involving 337 PD patients and 316 controls. One of these studies showed a significant decrease [30] and another a significant increase in PD patients [77], but the results of the meta-analysis showed non-significant differences between PD patients and controls (Figure 7d).

#### 3.4.6. Hair

Two of the three studies addressing hair manganese levels [30,79], and the pooled data from these studies, involving 150 PD patients and 56 controls (Table 2 and Supplementary Table S1, Figure 7e), showed a significant increase in PD patients.

## 4. Discussion

Together with iron, other transition metals such as copper, zinc, and manganese seem to play a role in the generation of free radicals and oxidative stress contributing to the neuronal degeneration in the substantia nigra pars compacta in the PD brain. In this review and meta-analysis, we have tried to establish the status of these transition metals and ceruloplasmin (the main copper-related protein) in patients with PD.

Copper plays several biological roles, including its involvement in iron homeostasis and its function as a cofactor for numerous metalloproteins and metalloenzymes, such as ceruloplasmin, Cu/Zn superoxide-dismutase, cytochrome c oxidase, and dopamine beta hydroxylase. In addition, it intervenes in the biosynthesis of neurotransmitters and peptide hormones, and in the protection against free radicals [102]. In the current meta-analysis, we have shown a significant decrease in copper concentrations in the substantia nigra, caudate nuclei, putamen, globus pallidus medialis and lateralis, and cerebellum of patients with PD compared to controls. It is likely that copper levels were also decreased in other brain areas, according to the results of some individual studies, but these data would need to be replicated. We have also found a non-significant trend towards increased levels of copper in CSF and towards decreased copper levels in serum/plasma (according to the random effects model, which is more reliable than the common effect model, although according to which these changes were significant), as well as increased copper levels in the whole blood of patients with PD compared to controls, while copper levels in urine and hair did not differ significantly between the two study groups.

Reduced copper levels in the substantia nigra should hypothetically result in an impairment of defences against oxidative processes, leading to increased oxidative stress, mitochondrial dysfunction and increased neuronal vulnerability. Copper deficiency seems to alter the expression of key proteins in iron metabolism and is associated with dysfunction in iron export and iron accumulation [103]; increased iron in the substantia nigra is a well demonstrated feature in PD. In addition, it has been shown that copper is able to bind α-synuclein with high affinity, consequently promoting its aggregation, and this binding would lead to a decrease in copper concentration [104,105]. Interestingly, it has been shown that significant amounts of Fe, Cu and Zn are bound to neuromelanin in the neurons of human putamen, premotor cortex, cerebellum, and substantia nigra in normal subjects [106], and vulnerability of the dopaminergic neurons in the substantia nigra seems to be related to their neuromelanin content [107]. Substantia nigra and locus coeruleus (the regions that have the highest neuronal loss in PD), that contain a high amount of neuromelanin bound to these metals, show age-related changes in copper and zinc concentrations (especially a decrease in copper concentrations in locus coeruleus) [108].

In agreement with our study, a previous meta-analysis showed decreased copper concentrations in the substantia nigra of PD patients, but in this study, the authors did not analyze copper concentrations in other brain areas [7]. Scolari-Grotto et al. [8], in a meta-analysis that only included articles published between 2011 and 2022 (therefore excluding several important pioneer studies), reported “decreased brain copper levels”, but in such a study, the authors combined the concentrations of copper in several brain areas. Two previous meta-analyses, with a lower number of studies analyzed and a lower sample size than the current meta-analysis, did not find significant differences in CSF copper levels between PD patients and controls [3,7,9]. These studies also described a non-significant trend towards lower serum/plasma copper levels in PD patients [3,7,9], similar to the results obtained by the random effects model in the current study. Scolari-Grotto et al. [8], with a lower sample size, also described decreased serum/plasma copper in PD patients. To our knowledge, concentrations of copper in whole blood (increased in this study), urine (similar to those of controls), and hair (similar to those of controls) in PD patients have not been addressed previously in other meta-analyses.

The ferroxidase enzyme ceruloplasmin, which is the main copper-carrying protein in the blood, and which plays an important role in iron metabolism, has also been found to be significantly decreased in the serum/plasma from PD patients compared to controls in the current study. This result is in disagreement with that of Wei et al. [3], who, in a meta-analysis with a considerably smaller sample size, did not find significant differences between patients with PD and controls. Data from studies on the brain [16] and CSF [23,81] are insufficient to draw valid conclusions. Ceruloplasmin, acting as ferroxidase, converts Fe^2+^ to less toxic Fe^3+^, and it has been shown that low ceruloplasmin concentration and activity in serum seems to be correlated to the high iron amounts in the substantia nigra [109].

Biological functions of zinc include, among many others, direct or indirect antioxidant actions, acting as a cofactor of Cu/Zn superoxide-dismutase, intervening in the synthesis of metallothioneins and metalloproteins/metalloenzymes, increasing glutathione peroxidase activity, regulation of apoptosis and inflammatory processes, participation in protein, RNA and DNA synthesis and in DNA replication, and being a cofactor of many enzymatic reactions and transcription factors [102]. The current meta-analysis showed increased zinc levels in the cerebellum and *globus pallidus lateralis* of PD patients with the common effect models (which was not confirmed with the more reliable random effect models), with non-significant differences between PD patients and controls in other brain regions, including the substantia nigra (in agreement with Genoud et al. [7]), caudate, and putamen. In agreement with previous meta-analyses [3,7,9], our results did not find significant differences in CSF levels between PD patients and controls, although there was a trend towards increased CSF levels in PD patients. Serum/plasma zinc concentrations were significantly lower in PD patients, as previously reported by other meta-analytic studies with smaller sample sizes [3,7,9,10,11]. In addition, we have shown, apparently for the first time, increased whole blood and hair zinc levels in PD patients and similar urine zinc levels in PD patients and controls. Lower systemic (serum/plasma) zinc and increased zinc levels in blood cells and hair in PD patients could reflect redistribution to tissues or altered zinc homeostasis [110], but decreased serum/plasma zinc levels could be a consequence of oxidative stress processes as well [26]. In addition. it has been shown that the binding of zinc to α-synuclein promotes its aggregation toward accelerated amyloid formation [111], a mechanism that could be involved in the formation of structures similar to Lewy bodies—the hallmark of PD.

Manganese acts as a cofactor of many metalloproteins/metalloenzymes—such as Mn-superoxide dismutase (oxidative stress), phosphoenolpyruvate carboxykinase (gluconeogenesis), pyruvate carboxylase (gluconeogenesis and lipogenesis), acetyl-CoA decarboxylase (fatty acid synthesis), glutamine synthetase (metabolism of glutamate to glutamine)—plays an important role in the homeostasis of iron, copper, zinc, and calcium, and has effects on several neurotransmitter systems, including dopaminergic (reduced dopamine levels by reduction in tyrosine hydroxylase activity), GABAergic, glutamatergic, and cholinergic [102]. Data from studies on the brain [14,20] were scarce and did not lead to valid conclusions. According to the current meta-analysis, CSF, serum/plasma, whole blood and urine manganese concentrations were similar in PD patients and controls, and were significantly increased in the hair of PD patients. Our results regarding CSF manganese levels were in agreement with those of previous meta-analyses with smaller sample sizes [3,7,112]. Similarly to our study, the other two meta-analyses did not find significant differences between PD patients and controls in serum/plasma manganese levels [3,7], while another one described this parameter as increased in PD patients [112]. To our knowledge, our meta-analysis is the first addressing urine and hair manganese concentrations in PD. Increased manganese in hair could be related to systemic manganese imbalance in PD patients, possibly reflecting altered manganese metabolism or excretion.

To date, there are only a few studies on the levels of copper, ceruloplasmin, zinc, and manganese in experimental models of PD. Interestingly, in the model of PD induced by 6-hydroxydopamine in rats, the concentrations of copper, zinc, and manganese in the substantia nigra, globus pallidus, putamen, and amygdala increased after injection of this toxin in the medial forebrain bundle [113], while mRNA and protein expressions of CP in the substantia nigra decreased compared to controls [114]. Administration of 1-methyl-4-phenyl-tetrahydropyridine (MPTP) to rodents caused an increase in copper concentrations in the rostral periventricular zone and a mild decrease in the interpeduncular nucleus, but no changes in the substantia nigra [115]. Finally, administration of the copper chelator D-penicillamine was not able to prevent MPTP-induced dopamine depletion in mice [116].

The main strength of our study would be the significant sample size, especially concerning studies conducted in serum/plasma and CSF, which would have considerable statistical power. However, as the main limitation, there was substantial variability among the different studies included, likely related both to methodological aspects and to the selection criteria used for patients with PD, which led to considerable heterogeneity in the pooled results. Table 3 summarizes the main advantages, limitations, and sensitivity of the different methods used for the determination of copper, zinc, and manganese levels in the studies included in the meta-analyses [117,118,119,120,121,122,123]. In any case, the exclusion of studies without a control group ensures that, for each of the studies included in the meta-analysis, the experimental conditions for patients with PD and the controls are the same. In addition, to exclude a possible influence of studies with data collected close to 1966, we performed a subgroup analysis including only those studies published after 1985 which showed the same results.

## 5. Conclusions

Taking these limitations into account, the main findings of this meta-analysis were (1) the decrease in copper concentrations in many brain regions, including the substantia nigra and basal ganglia; (2) the decrease in ceruloplasmin levels in the serum/plasma of PD patients; (3) the decrease in serum/plasma and increase in whole blood and hair zinc concentrations in PD patients; and (4) the increase of manganese levels in the hair of PD patients. Globally, these results could suggest a possible role of global ceruloplasmin (with copper near statistical significance) and zinc deficiency in the risk for PD (perhaps related to the role of these metals in oxidative stress), despite a previous meta-analysis not showing a lower dietary intake of copper and zinc in PD patients [124].

## Figures and Tables

**Table 1:** **Search strategy. The numbers in the cell correspond to the items found in the search performed with PubMed and Web of Science (Core Collection) on** **29 November 2025**.

	Copper	Ceruloplasmin	Zinc	Manganese
**Brain**	346	93	298	485
**Cerebrospinal fluid**	30	23	19	22
**Serum**	86	59	56	50
**Plasma**	70	22	49	67
**Blood**	117	48	95	132
**Urine**	20	7	12	30
**Hair**	14	1	7	13

**Table 2:** **Studies assessing brain, CSF, serum/plasma, whole blood, urine, and hair concentrations of copper, ceruloplasmin, zinc, and manganese in patients with Parkinson’s disease and controls. Data from column “main results (2)” express degree of increase (+) or decrease (−) from 1 to 3, with 0 meaning differences are non-significant. For details see** **Supplementary Table S1**.

	Copper				
Tissue	Author, Year [Ref]	Method	Main Results	Main Results (2)	Comments/ Observations
**Brain**	Riederer et al., 1989 [12]	Atomic absorption spectrophotometry	Significant increase in the raphe plus reticular formation and red nucleus in PD brains, and non-significant differences in other brain areas, including substantia nigra, caudate, putamen, and globus pallidus.	3− for raphe, reticular formation and red nucleus	Data obtained as estimated from a graphic.
	Dexter et al., 1989 [13]	Inductively coupled plasma spectroscopy	Significant decrease in substantia nigra from PD brains (34–45%). Non-significant differences between PD and controls in cerebellum, cerebral cortex, caudate nucleus, putamen, and globus pallidus.	3− for substantia nigra	Data obtained as estimated from a graphic.
	Uitti et al., 1989 [14]	Atomic emission spectroscopy and atomic absorption spectrophotometry	Significant decrease in the substantia nigra from PD brains, and non-significant differences with controls in the frontal cortex, caudate nucleus, and cerebellum.	3− for substantia nigra	
	Dexter et al., 1991 [15]	Inductively coupled plasma spectroscopy	Significant decrease in the substantia nigra from PD brains. Non-significant differences between PD and controls in cerebellum, cerebral cortex (Brodmann area 10), caudate nucleus, putamen, and globus pallidus.	3− for substantia nigra	
	Loeffler et al., 1996 [16]	Flame atomic absorption spectrophotometry	Significant decrease in caudate from PD brains. Non-significant differences between PD and elderly controls in the substantia nigra, putamen, and frontal cortex.	2− for caudate	Data expressed in ng/μg of protein. Not included in meta-analysis.
	Wypijewska et al., 2010 [17]	Electrothermal atomic absorption spectrometry	Significant decrease in the substantia nigra from PD patients.	3− for caudate	
	Szczerbowska-Boruchoswska et al., 2012 [18]	Synchrotron radiation-based X-ray fluorescence (SRXRF)	Non-significant differences between PD and controls.	0	Data expressed in Cu mass fraction in the substantia nigra. Not included in the meta-analysis.
	Davies et al., 2014 [19]	Synchrotron radiation x-ray fluorescence microscopy (SRXFM) and particle-induced x-ray emission (PIXE) microscopy	Significant decrease in the substantia nigra and locus ceruleus from PD patients. Non-significant differences between PD and controls in the occipital cortex.	3− for substantia nigra and locus ceruleus	
	Gardner et al., 2017 [20]	Inductively coupled plasma spectroscopy	Non-significant differences between PD and controls in the olfactory bulb and olfactory tract.	0	
	Genoud et al., 2017 [21]	Inductively coupled plasma spectroscopy	Significant decrease in the substantia nigra from PD patients (54%). Non-significant differences between PD and controls in the occipital cortex and fusiform gyrus.	3− in substantia nigra	Full data not available. Not included in the meta-analysis.
**CSF**	Mindadse & Tschikowani, 1967 [22]	Not available	Not available		Unable to get this article; abstract not available.
	Campanella et al., 1973 [23]	Colorimetric method	Non-significant differences between PD patients and controls.	0	
	Pall et al., 1987 [24]	Electrochemical atomization/atomic absorption spectrophotometry	Significant increase in PD patients compared to controls.	3+	Controls were subjects with neurological diseases who had a clinical indication for lumbar puncture. Estimation from a graphic.
	Gazzaniga et al., 1992 [25]	Atomic absorption spectrophotometry with electrothermal atomization	Non-significant differences between PD patients and controls.	0	
	Jiménez-Jiménez et al., 1998 [26]	Flame atomic absorption spectrophotometry	Non-significant differences between PD patients and controls.	0	Controls were subjects with neurological diseases who had a clinical indication for lumbar puncture.
	Boll et al., 1999 [27]	Atomic absorption spectrophotometry	Non-significant differences between PD patients and controls.	0	
	Forte et al., 2004 [28]	Inductively coupled plasma atomic emission spectrometry	Non-significant differences between PD patients and controls.	0	
	Qureshi et al., 2006 [29]	Atomic absorption spectrophotometry with an electrothermal atomizer	Non-significant differences between PD patients and controls.	0	
	Bocca et al., 2006 [30]	Inductively coupled plasma atomic emission spectrometry	Non-significant differences between PD patients and controls.	0	Data obtained as estimated from a graphic.
	Alimonti et al., 2007 [31]	Inductively coupled plasma mass spectrometry and sector field inductively coupled Plasma Mass Spectrometry	Non-significant differences between PD patients and controls.	0	
	Boll et al., 2008 [32]	Graphite furnace atomic absorption spectrophotometry	Significant increase in PD patients compared to controls.	3+	Measurement of free (and not total) copper. This article was excluded from meta-analysis.
	Hozumi et al., 2011 [33]	Inductively coupled plasma spectroscopy	Significant increase in PD patients.	3+	
	Sanyal et al., 2016 [34]	Atomic absorption spectrophotometry and flame atomic absorption spectrophotometry	Non-significant differences between PD patients and controls.	0	
	Maass et al., 2018 [35]	Inductively coupled plasma-sector field mass spectrometry	Non-significant differences between PD patients and controls.	0	
	Willkommen et al., 2018 [36]	Size-exclusion chromatography hyphenated to inductively coupled plasma mass spectrometry (SEC-ICP-MS)	Non-significant differences between PD patients and controls.	0	
	Dušek et al., 2025 [37]	Inductively coupled plasma spectroscopy	Significant decrease in PD patients.	1−	
**Serum/Plasma**	Mindadse & Tschikowani, 1967 [22]	Not available	Not available		Unable to get this article; abstract not available.
	Kanabrocki et al., 1967 [38]	RCL 512-Channel Analyzer (an instrument used in nuclear and radiation spectroscopy)	Non-significant differences between PD patients and controls.	0	
	Chitre & Punekar, 1970 [39]	Iodometric redox titration	Significant increase in PD patients compared to controls.	3+	
	Campanella et al., 1973 [23]	Colorimetric methods	Non-significant differences between PD patients and controls.	0	
	Jiménez-Jiménez et al., 1992 [40]	Flame atomic absorption spectrophotometry	Non-significant differences between PD patients and controls.	0	
	Abbott et al., 1992 [41] (plasma)	Atomic absorption spectrophotometry	Non-significant differences between PD patients and controls.		
	Jiménez-Jiménez et al., 1998 [26]	Flame atomic absorption spectrophotometry	Non-significant differences between PD patients and controls.	0	Controls were subjects with neurological diseases who had a clinical indication for lumbar puncture. PD patients and controls were not the same as in [CMZ-14].
	Tórsdóttir et al., 1999 [42] (plasma)	Atomic absorption spectrophotometry	Non-significant differences between PD patients and controls.	0	
	Kocatürk et al., 2000 [43] (plasma)	Atomic absorption spectrophotometry	Significant increase in PD patients compared to controls.		
	Forte et al., 2004 [28]	Inductively Coupled Plasma Atomic Emission Spectrometry	Significant decrease in PD patients compared to controls.	2+	
	Hegde et al., 2004 [44]	Inductively Coupled Plasma Atomic Emission Spectrometry	Significant increase in PD patients compared to controls.	2−	
	Qureshi et al., 2006 [29]	Atomic absorption spectrophotometry with an electrothermal atomizer	Non-significant differences between PD patients and controls.	0	
	Bocca et al., 2006 [30]	Inductively Coupled Plasma Atomic Emission Spectrometry	Significant decrease in PD patients compared to controls.	1−	
	Alimonti et al., 2007 [45]	Inductively Coupled Plasma Atomic Emission Spectrometry	Non-significant differences between PD patients and controls.	0	
	Squitti et al., 2007 [46]	Graphite furnace atomic absorption.	Non-significant differences between PD patients and controls.	0	
	Gellein et al., 2008 [47]	High Resolution Inductively Coupled Plasma Atomic Emission Spectrometry	Non-significant differences between PD patients and controls.	0	
	Bharucha et al., 2009 [48]	Not specified	Significant decrease in PD patients compared to controls.	2−	
	Nikam et al., 2009 [49] (plasma)	Atomic absorption spectrophotometry	Significant decrease in PD patients compared to controls.	2−	
	Fukushima et al., 2010, 2011, 2013, 2014 [50,51,52,53]	Inductively Coupled Plasma Atomic Emission Spectrometry	Non-significant differences between PD patients and controls.	0	
	Arnal et al., 2010 [54] (plasma)	Atomic absorption spectrophotometry	Non-significant differences between PD patients and controls.	0	
	Henkin et al., 2010 [55]	Atomic absorption spectrophotometry	Lack of a control group.		Lack of a control group
	Baillet et al., 2010 [56]	Atomic absorption Spectrophotometry	Non-significant differences between PD patients and controls.	0	
	Ahmed & Santosh 2010 [57]	Inductively Coupled Plasma Atomic Emission Spectrometry and atomic absorption spectroscopy	Significant increase in PD patients compared to controls.	2+	
	Ling & Bidhayasiri 2011 [58]	Not specified	Non-significant differences between PD patients and controls.	0	Data not given
	McIntosh et al., 2012 [59] (plasma)	Monochromatic X-ray fluorescence spectrometry	Non-significant differences between PD patients and controls.	0	
	Mariani et al.,2013 [60]	Not specified	Non-significant differences between PD patients and controls.	0	Data not available in the Supplementary Material.
	Younes-Mehnni et al., 2013 [61]	Plasma atomic absorption spectrophotometry	Significant decrease in PD patients compared to controls.	2−	
	Zhao et al., 2013 [62] (plasma)	Zeeman atomic absorption spectroscopy with a graphite tube atomizer	Significant decrease in PD patients compared to controls.	2−	
	Kumudini et al. 2014 [63] (plasma)	Inductively Coupled Plasma Atomic Emission Spectrometry	Significant increase in PD patients compared to controls.	2+	
	Meamar et al., 2016 [64] (plasma)	Colorimetric method	Lack of a control group.		Lack of a control group.
	Sanyal et al., 2016 [34]	Atomic absorption spectrophotometry and flame atomic absorption spectrophotometry	Significant decrease in PD patients compared to controls.	2−	
	Song et al., 2017 [65]	Immunoassay	Non-significant differences between PD patients and controls.	0	
	Gangania et al., 2017 [66]	Colorimetric method	Significant decrease in PD patients compared to controls.	2−	
	Karpenko et al., 2018 [67] (plasma)	Atomic absorption spectrophotometry	Significant decrease in PD patients compared to controls.	3−	
	Ilyechova et al., 2018 [68]	Graphite furnace atomic absorption spectrometry	Significant decrease in PD patients compared to controls.	3−	
	Kim et al., 2018 [69]	Inductively Coupled Plasma Atomic Emission Spectrometry	Significant decrease in PD patients compared to controls.	1−	
	Ajsuvakova et al., 2020 [70]	Inductively Coupled Plasma Atomic Emission Spectrometry	Non-significant differences between PD patients and controls.		
	Fattah et al., 2020 [71]	Graphite furnace atomic absorption spectrophotometry	Significant decrease in PD patients compared to controls.	2−	
	Barmaki et al., 2021 [72]	Graphite furnace atomic absorption spectrophotometry	Significant decrease in PD patients compared to controls.	2−	
	Tripathi et al., 2021 [73]	Spectrophotometery immunoturbidimetry	Significant decrease in PD patients compared to controls.	3−	
	Lee et al., 2023 [74]	Inductively coupled plasma atomic emission spectrometry	Lack of a control group. Non-significant difference between PD patients with and without dementia.		Lack of a control group.
	Kim et al., 2023 [75]	Inductively coupled plasma atomic emission spectrometry	Lack of a control group. Non-significant difference between PD patients with and without levodopa-induced dyskinesia.		Lack of a control group.
	Melek et al., 2023 [76]	Atomic emission spectrometry	Non-significant differences between PD patients and controls.	0	
**Whole Blood**	Bocca et al., 2006 [30]	Inductively coupled plasma atomic emission spectrometry	Significant increase in PD patients compared to controls.	2+	
	McIntosh et al., 2012 [59]	Monochromatic X-ray fluorescence spectrometry	Non-significant differences between PD patients and controls.	0	
	Dušek et al., 2025 [37]	Inductively coupled plasma spectroscopy	Non-significant differences between PD patients and controls.	0	
**Urine**	McIntosh et al., 2012 [59]	Monochromatic X-ray fluorescence spectrometry	Non-significant differences between PD patients and controls.	0	
	Forte et al., 2004 [28]	Inductively coupled plasma atomic emission spectrometry	Non-significant differences between PD patients and controls.	0	
	Bocca et al., 2006 [30]	Inductively coupled plasma atomic emission spectrometry	Non-significant differences between PD patients and controls.	0	Data obtained as estimated from a graphic.
	Fukushima et al., 2011, 2013, 2014 [51,52,53]	Atomic absorption spectrometry	Significant increase in PD patients compared to controls.	1+	
	Ajsuvakova et al., 2020 [70]	Inductively coupled plasma atomic emission spectrometry	Non-significant differences between PD patients and controls.	0	
	Shan et al., 2025 [77]	Inductively coupled plasma atomic emission spectrometry	Non-significant differences between PD patients and controls.	0	
**Hair**	Forte et al., 2005 [78]	Inductively coupled plasma atomic emission spectrometry	Non-significant differences between PD patients and controls.	0	
	Bocca et al., 2006 [30]	Inductively coupled plasma atomic emission spectrometry	Non-significant differences between PD patients and controls	0	Data obtained as estimated from a graphic.
	Stefano et al., 2016 [79]	Inductively coupled plasma atomic emission spectrometry	Non-significant differences between PD patients and controls.	0	
	Ajsuvakova et al., 2020 [70]	Inductively coupled plasma atomic emission spectrometry	Significant increase in PD patients compared to controls.	2+	
	**Ceruloplasmin**				
**Tissue**	**Author, Year [Ref]**	**Method**	**Main Results**	**Main Results (2)**	**Comments/** **Observations**
**Brain**	Loeffler et al., 1996 [16]	Enzyme-linked immunosorbent assay (ELISA)	Significant increase in PD substantia nigra, frontal, temporal, and parietal cortices, and hippocampus; and non-significant differences with elderly controls in caudate, putamen, and cerebellum.	1+ in mentioned brain areas	Data given as corrected by proteins instead of g of tissue.
	Loeffler et al., 2001 [80]	Immunocytochemical staining with avidin-biotin-peroxidase complex	Non-significant differences in ceruloplasmin immunoreactivity in substantia nigra, caudate, frontal and parietal cortices, parahippocampus, subiculum, and CA1 region.	0	Data given in immunoreactivity for ceruloplasmins, not in concentrations, data not included.
**CSF**	Campanella et al., 1973 [23]	Colorimetric methods	Non-significant differences between PD patients and controls.	0	
	Loeffler et al., 1994 [81]	Enzyme-linked immunosorbent assay (ELISA)	Non-significant differences between PD patients and controls.	0	
**Serum**	Campanella et al., 1973 [23]	Colorimetric methods	Non-significant differences between PD patients and controls.	0	
	Jiménez-Jiménez et al., 1992 [40]	Nephelometry immunoassay	Non-significant differences between PD patients and controls.	0	
	Molina-Arjona et al., 1999 [82]	Nephelometry immunoassay	Non-significant differences between PD patients and controls.	0	
	Tórsdóttir et al., 1999 [42] (plasma)	Nephelometry immunoassay	Significant decrease in PD patients compared to controls.	0	Non-significant changes when recalculated.
	Tórsdóttir et al., 2006 [83]	Nephelometry immunoassay	Significant decrease in PD patients compared to controls.	0	Non-significant changes when recalculated.
	Squitti et al., 2007 [46]	Immunoturbidimetric assay	Non-significant differences between PD patients and controls.	0	
	Bharucha et al., 2009 [48]	Nephelometric immu-neturbidimetric assay	Significant decrease in PD patients compared to controls.	2−	
	Nikam et al., 2009 [49] (plasma)	Colorimetric method	Significant decrease in PD patients compared to controls.		Concentrations expressed as Units/L, Conversion to mg/dL not available. Data not included.
	Arnal et al., 2010 [54] (plasma)	Atomic absorption spectrophotometry	Non-significant differences between PD patients and controls.	0	
	Jin et al., 2011 [84]	Nephelometry immunoassay	Significant decrease in PD patients compared to controls.	1−	
	Ling & Bidhayasiri 2011 [58]	Immunoturbidimetric assay with specific antiserum	Significant decrease in PD patients compared to controls.	0	Non-significant changes when recalculated.
	Zhao et al. 2014 [85]	Nephelometry immunoassay	Significant decrease in PD patients compared to controls.	2−	
	Song et al., 2017 [65]	Immunoassay	Significant decrease in PD patients compared to controls.	1−	
	Gangania et al., 2017 [66]	Turbidimetric assay	Non-significant differences between PD patients and controls.	0	
	Karpenko et al., 2018 [67] (plasma)	Atomic absorption spectrophotometry	Significant decrease in PD patients compared to controls.	1−	
	Ilyechova et al., 2018 [68]	Spectrophotometric method with p-phenylenediamine	Significant decrease in PD patients compared to controls.	1−	
	Xu et al., 2018 [86]	Immunoturbidimetric assay	Non-significant differences between PD patients and controls.	0	
	Si et al., 2018 [87] (plasma)	Immunoturbidimetric assay	Non-significant differences between PD patients and controls.	0	
	Bakeberg et al., 2020 [88]	Immunoturbidimetric assay	Lack of a control group. High impulsivity in PD patients with high compared with those with low ceruloplasmin levels.		Lack of a control group.
	Tripathi et al., 2021 [73]	Spectrophotometery immunoturbidimetry	Non-significant differences between PD patients and controls.	0	
	**Zinc**				
**Tissue**	**Author, Year [Ref]**	**Method**	**Main Results**	**Main Results (2)**	**Comments/** **Observations**
**Brain**	Riederer et al., 1989 [12]	Atomic absorption spectrophotometry	Significant increase in the raphe plus reticular formation in PD brains, and non-significant differences in other brain areas, including substantia nigra, caudate, putamen, and globus pallidus.	0	Data obtained as estimated from a graphic. Non-significant changes when recalculated.
	Dexter et al., 1989 [13]	Inductively coupled plasma spectroscopy	Significant increase in substantia nigra from PD brains (50–54%) and in lateral putamen (18–35%). Non-significant differences between PD and controls in cerebellum, cerebral cortex, caudate nucleus, medial putamen, and globus pallidus.	3+ for substantia nigra, 2+ for lateral putamen	Data obtained as estimated from a graphic.
	Uitti et al., 1989 [14]	Atomic emission spectroscopy and atomic absorption spectrophotometry	Non-significant differences between PD and controls in the frontal cortex, caudate nucleus, and cerebellum.	0	
	Hirsch et al., 1991 [89]	X-ray microanalysis	Non-significant differences between PD and controls in the substantia nigra (cells positive or negative for neuromelanin) and central grey substance.		Data from the control group not available.
	Dexter et al., 1991 [15]	Inductively coupled plasma spectroscopy	Significant increase in substantia nigra from PD brains. Non-significant differences between PD and controls in cerebellum, cerebral cortex (Brodmann area 10), caudate nucleus, putamen, and globus pallidus.	0	Non-significant changes when recalculated.
	Mann et al., 1994 [90]	Inductively coupled plasma spectroscopy	Non-significant differences between PD patients and controls in the substantia nigra.	0	
	Popescu et al., 2009 [91]	Rapid-scanning X-ray fluorescence mapping	Decrease fluorescence in substantia nigra, caudate, putamen, internal and external globus pallidus, inferior colliculus, white matter, internal capsule, grey matter, and optic tract.	1− for all mentioned brain areas	Data given in normalized fluorescence
	Szczerbowska-Boruchoswska et al., 2012 [18]	Synchrotron radiation-based X-ray fluorescence	Significant increase in Zn mass fraction in the substantia nigra from PD patients.	1+	
	Davies et al., 2014 [19]	Particle-induced X-ray emission (PIXE) microscopy	Non-significant differences between PD and controls in the substantia nigra, locus ceruleus, and occipital cortex.	0	
	Gardner et al., 2017 [20]	Inductively coupled plasma spectroscopy	Non-significant differences between PD and controls in the olfactory bulb and olfactory tract.	0	
	Genoud et al., 2017 [21]	Inductively coupled plasma spectroscopy	Non-significant differences between PD and controls in the substantia nigra, occipital cortex, and fusiform gyrus.	0	Full data not available
**CSF**	Mindadse & Tschikowani, 1967 [22]	Not available	Not available		Unable to get this article; abstract not available.
	Jiménez-Jiménez et al., 1998 [26]	Flame atomic absorption spectrophotometry	Significant decrease in PD patients.	3−	Controls were subjects with neurological diseases who had a clinical indication for lumbar puncture.
	Forte et al., 2004 [28]	Inductively coupled plasma atomic emission spectrometry	Significant decrease in PD patients.	1−	
	Qureshi et al., 2006 [29]	Atomic absorption spectrophotometry with an electrothermal atomizer	Non-significant differences between PD patients and controls.	0	
	Bocca et al., 2006 [30]	Inductively coupled plasma atomic emission spectrometry	Non-significant differences between PD patients and controls.	0	Data obtained as estimated from a graphic.
	Alimonti et al., 2007 [31]	Inductively Coupled Plasma Mass Spectrometry and Sector Field Inductively Coupled Plasma Mass Spectrometry	Non-significant differences between PD patients and controls.	0	
	Hozumi et al., 2011 [33]	Inductively coupled plasma spectroscopy	Significant increase in PD patients.	3+	
	Sanyal et al., 2016 [34]	Atomic absorption spectrophotometry and flame atomic absorption spectrophotometry	Significant decrease in PD patients compared to controls.	1−	
	Maass et al., 2018 [35]	Inductively coupled plasma-sector field mass spectrometry	Non-significant differences between PD patients and controls.	0	
	Willkommen et al., 2018 [36]	Size-exclusion chromatography hyphenated to inductively coupled plasma mass spectrometry (SEC-ICP-MS)	Non-significant differences between PD patients and controls.	0	
	Dušek et al., 2025 [37]	Inductively coupled plasma spectroscopy	Non-significant differences between PD patients and controls.	0	
**Serum**	Mindadse & Tschikowani, 1967 [22]	Not available	Not available		Unable to get this article; abstract not available.
	Jiménez-Jiménez et al., 1992 [40]	Flame atomic absorption spectrophotometry	Non-significant differences between PD patients and controls.	0	
	Abbott et al., 1992 [41] (plasma)	Atomic absorption spectrophotometry	Significant decrease in PD patients.	3−	
	Jiménez-Jiménez et al., 1998 [26]	Flame atomic absorption spectrophotometry	Non-significant differences between PD patients and controls.	0	Controls were subjects with neurological diseases who had a clinical indication for lumbar puncture. PD patients and controls were not the same as in [40].
	Kocatürk et al., 2000 [43] (plasma)	Atomic absorption spectrophotometry	Non-significant differences between PD patients and controls.	0	
	Forte et al., 2005 [78]	Inductively coupled plasma atomic emission spectrometry	Non-significant differences between PD patients and controls.	0	
	Hegde et al., 2004 [44]	Inductively coupled plasma atomic emission spectrometry	Significant decrease in PD patients compared to controls.	2−	
	Qureshi et al., 2006 [29]	Atomic absorption spectrophotometry with an electrothermal atomizer	Non-significant differences between PD patients and controls.	0	
	Bocca et al., 2006 [30]	Inductively coupled plasma atomic emission spectrometry	Non-significant differences between PD patients and controls.	0	Data obtained as estimated from a graphic.
	Alimonti et al., 2007 [31]	Inductively coupled plasma atomic emission spectrometry	Non-significant differences between PD patients and controls.	0	
	Squitti et al., 2007 [46]	Graphite furnace atomic absorption.	Non-significant differences between PD patients and controls.	0	
	Gellein et al., 2008 [47]	High resolution inductively coupled plasma atomic emission spectrometry	Non-significant differences between PD patients and controls.	0	
	Nikam et al., 2009 [49]	Atomic absorption spectrophotometry	Significant decrease in PD patients compared to controls.	2−	
	Fukushima et al., 2010, 2011, 2013, 2014 [50,51,52,53]	Inductively coupled plasma atomic emission spectrometry	Non-significant differences between PD patients and controls.	0	
	Henkin et al., 2010 [55]	Atomic absorption spectrophotometry	Lack of a control group.		Lack of a control group.
	Baillet et al., 2010 [56]	Atomic absorption spectrophotometry	Significant decrease in PD patients.	0	
	Ahmed & Santosh 2010 [57]	Inductively coupled plasma atomic emission spectrometry and atomic absorption spectroscopy	Significant decrease in PD patients compared to controls.	0	
	Brewer et al., 2010 [92]	Atomic absorption spectrophotometry	Significant decrease in PD patients.	1−	
	McIntosh et al., 2012 [59] (plasma)	Monochromatic X-ray fluorescence spectrometry	Non-significant differences between PD patients and controls.	0	
	Younes-Mehnni et al., 2013 [61]	Plasma atomic absorption spectrophotometry	Significant decrease in PD patients compared to controls.	0	Non-significant differences when recalculated.
	Zhao et al., 2013 [62] (plasma)	Fast sequential atomic absorption spectroscopy	Significant decrease in PD patients compared to controls.	2−	
	Verma et al., 2015 [93]	Inductively coupled plasma atomic emission spectrometry	Significant decrease in PD patients.	3−	
	Meamar et al., 2016 [64] (plasma)	Colorimetric method	Lack of a control group.		
	Sanyal et al., 2016 [34]	Atomic absorption spectrophotometry and flame atomic absorption spectrophotometry	Non-significant differences between PD patients and controls.	0	
	Kim et al., 2018 [69]	Inductively coupled plasma atomic emission spectrometry	Non-significant differences between PD patients and controls.	0	
	Ajsuvakova et al., 2020 [70]	Inductively coupled plasma atomic emission spectrometry	Non-significant differences between PD patients and controls.	0	
	Fattah et al., 2020 [71]	Graphite furnace atomic absorption spectrophotometry	Significant decrease in PD patients compared to controls.	2−	
	Barmaki et al., 2021 [72]	Graphite furnace atomic absorption spectrophotometry	Significant decrease in PD patients compared to controls.	2−	
	Matsuyama et al., 2021 [94]	Not specified	Lack of a control group.		
	Lee et al., 2023 [74]	Inductively coupled plasma atomic emission spectrometry	Lack of a control group. Increase in patients with PD who developed dementia compared to PD patients without dementia during the follow-up period.		Lack of a control group.
	Kim et al., 2023 [75]	Inductively coupled plasma atomic emission spectrometry	Lack of a control group. Decrease in patients with PD who developed levodopa-induced dyskinesia compared to PD patients without this adverse effect during the follow-up period.		Lack of a control group.
	Melek et al., 2023 [76]	Atomic emission spectrometry	Significant decrease in PD patients.	2−	
**Whole Blood**	Bocca et al., 2006 [30]	Inductively coupled plasma atomic emission spectrometry	Significant increase in PD patients.	1+	
	McIntosh et al., 2012 [59]	Monochromatic X-ray fluorescence spectrometry	Non-significant differences between PD patients and controls.	0	
	Dušek et al., 2025 [37]	Inductively coupled plasma spectroscopy	Significant increase in PD patients.	1+	
**Urine**	McIntosh et al., 2012 [59]	Monochromatic X-ray fluorescence spectrometry	Non-significant differences between PD patients and controls.	0	
	Forte et al., 2004 [28]	Inductively coupled plasma atomic emission spectrometry	Non-significant differences between PD patients and controls.	0	
	Bocca et al., 2006 [30]	Inductively Coupled Plasma Atomic Emission Spectrometry	Non-significant differences between PD patients and controls.	0	Data obtained as estimated from a graphic.
	Brewer et al., 2010 [92]	Atomic absorption spectrophotometry	Significant increase in PD patients.	2+	Standard deviation values not available. Not included in the meta-analysis.
	Fukushima et al., 2011, 2013, 2024 [51,52,53]	Atomic absorption spectrometry	Non-significant differences between PD patients and controls.	0	
	Ajsuvakova et al., 2020 [79]	Inductively coupled plasma atomic emission spectrometry	Non-significant differences between PD patients and controls.	0	
	Shan et al., 2025 [77]	Inductively coupled plasma atomic emission spectrometry	Non-significant differences between PD patients and controls.	0	
**Hair**	Forte et al., 2004 [28]	Inductively coupled plasma atomic emission spectrometry	Non-significant differences between PD patients and controls.	0	
	Bocca et al., 2006 [30]	Inductively coupled plasma atomic emission spectrometry	Non-significant differences between PD patients and controls.	0	Data obtained as estimated from a graphic.
	Stefano et al., 2016 [79]	Inductively coupled plasma atomic emission spectrometry	Non-significant differences between PD patients and controls.	0	
	Dos Santos et al. 2018, 2019 [95,97]	Flame atomic absorption spectrophotometry	Significant increase in PD patients.	3+	
	Ajsuvakova et al., 2020 [70]	Inductively coupled plasma atomic emission spectrometry	Non-significant differences between PD patients and controls.	0	
	**Manganese**				
**Tissue**	**Author, Year [Ref]**	**Method**	**Main Results**	**Main Results (2)**	**Comments/** **Observations**
**Brain**	Larsen et al., 1981 [96]	Neutron activation analysis with radiochemical separation	Non-significant differences between PD patients (n = 2) and controls (n = 5) for 24 brain areas.	0	Not included in the meta-analysis. There are no statistical data.
	Dexter et al., 1989 [13]	Inductively coupled plasma spectroscopy	Significant decrease in medial putamen from PD brains (20%). Non-significant differences between PD and controls in the substantia nigra, cerebellum, cerebral cortex, caudate nucleus, lateral putamen, and globus pallidus.	1−	Data obtained as estimated from a graphic.
	Uitti et al., 1989 [14]	Atomic emission spectroscopy and atomic absorption spectrophotometry	Non-significant differences between PD and controls in the frontal cortex, caudate nucleus, and cerebellum.	0	
	Dexter et al., 1991 [15]	Inductively coupled plasma spectroscopy	Non-significant differences between PD and controls in the substantia nigra, cerebellum, cerebral cortex (Brodmann area 10), caudate nucleus, putamen, and globus pallidus. There was a 20% decrease in the medial putamen of PD.	0	
	Gardner et al., 2017 [20]	Inductively coupled plasma spectroscopy	Non-significant differences between PD and controls in the olfactory bulb and olfactory tract.	0	
	Genoud et al., 2017 [21]	Inductively coupled plasma spectroscopy	Non-significant differences between PD and controls in the substantia nigra, occipital cortex, and fusiform gyrus.	0	Full data not available.
**CSF**	Mindadse & Tschikowani, 1967 [22]	Not available	Not available		Unable to get this article; abstract not available.
	Pall et al., 1987 [24]	Electrochemical atomization/atomic absorption spectrophotometry	Non-significant differences between PD patients and controls.	0	Controls were subjects with neurological diseases who had a clinical indication for lumbar puncture. Estimation from a graphic.
	Gazzaniga et al., 1992 [25]	Atomic absorption spectrophotometry with electrothermal atomization	Non-significant differences between PD patients and controls.	0	
	Jiménez-Jiménez et al., 1998 [26]	Flame atomic absorption spectrophotometry	Non-significant differences between PD patients and controls.	0	Controls were subjects with neurological diseases who had a clinical indication for lumbar puncture.
	Forte et al., 2004 [28]	Inductively coupled plasma atomic emission spectrometry	Non-significant differences between PD patients and controls.	0	
	Bocca et al., 2006 [30]	Sector field inductively coupled plasma mass spectrometry	Non-significant differences between PD patients and controls.	0	
	Hozumi et al., 2011 [33]	Inductively coupled plasma spectroscopy	Significant increase in PD patients.	3+	
	Alimonti et al., 2007 [31]	Inductively coupled plasma mass spectrometry and sector field inductively coupled plasma mass spectrometry	Non-significant differences between PD patients and controls.	0	
	Sanyal et al., 2016 [34]	Atomic absorption spectrophotometry and flame atomic absorption spectrophotometry	Non-significant differences between PD patients and controls.	0	
	Maass et al., 2018 [35]	Inductively coupled plasma-sector field mass spectrometry	Non-significant differences between PD patients and controls.	0	
	Willkommen et al., 2018 [36]	Size-exclusion chromatography hyphenated to inductively coupled plasma mass spectrometry (SEC-ICP-MS)	Non-significant differences between PD patients and controls.	0	
	Dušek et al., 2025 [37]	Inductively coupled plasma spectroscopy	Non-significant differences between PD patients and controls.	0	
**Serum**	Mindadse & Tschikowani, 1967 [22]	Not available	Not available		Unable to get this article; abstract not available
	Kanabrocki et al., 1967 [38]	RCL 512-Channel Analyzer (an instrument used in nuclear and radiation spectroscopy)	Non-significant differences between PD patients and controls.	0	
	Jiménez-Jiménez et al., 1992 [98]	Flame atomic absorption spectrophotometry	Non-significant differences between PD patients and controls.	0	
	Jiménez-Jiménez et al., 1998 [26]	Flame atomic absorption spectrophotometry	Non-significant differences between PD patients and controls.	0	Controls were subjects with neurological diseases who had a clinical indication for lumbar puncture. PD patients and controls were not the same as in [CMZ-14].
	Forte et al., 2004 [28]	Inductively coupled plasma atomic emission spectrometry	Non-significant differences between PD patients and controls.	0	
	Hegde et al., 2004 [44]	Inductively coupled plasma atomic emission spectrometry	Significant increase in PD patients compared to controls.	3+	Mean ± SD for controls not given. Not included in the meta-analysis.
	Bocca et al., 2006 [30]	Sector field inductively coupled plasma mass spectrometry	Non-significant differences between PD patients and controls.	0	
	Alimonti et al., 2007 [45]	Inductively coupled plasma atomic emission spectrometry	Non-significant differences between PD patients and controls.	0	
	Gellein et al., 2008 [47]	High resolution inductively coupled plasma atomic emission spectrometry	Non-significant differences between PD patients and controls.	0	
	Ahmed & Santosh 2010 [57]	Inductively coupled plasma atomic emission spectrometry and atomic absorption spectroscopy	Significant increase in PD patients compared to controls.	1+	
	Kumudini et al. 2014 [63] (plasma)	Inductively coupled plasma atomic emission spectrometry	Non-significant differences between PD patients and controls.	0	
	Verma et al., 2016 [93]	Inductively Coupled Plasma Atomic Emission Spectrometry	Significant increase in PD patients.	2+	
	Sanyal et al., 2016 [34]	Atomic absorption spectrophotometry and flame atomic absorption spectrophotometry	Non-significant differences between PD patients and controls.	0	
	Ajsuvakova et al., 2020 [70]	Inductively coupled plasma atomic emission spectrometry	Non-significant differences between PD patients and controls.	0	
	Lee et al., 2023 [74]	Inductively coupled plasma atomic emission spectrometry	Lack of a control group. Non-significant difference between PD patients with and without dementia.		Lack of a control group.
	Kim et al., 2023 [75]	Inductively coupled plasma atomic emission spectrometry	Lack of a control group. Non-significant difference between PD patients with and without levodopa-induced dyskinesia.		Lack of a control group.
**Whole Blood**	Bocca et al., 2006 [30]	Sector field inductively coupled plasma mass spectrometry	Non-significant differences between PD patients and controls.	0	Data obtained as estimated from a graphic.
	Fukushima et al., 2010, 2011, 2013 [50,51,52]	Atomic absorption spectrometry	Significant increase in PD patients.	3+	
	Dušek et al., 2025 [37]	Inductively coupled plasma spectroscopy	Non-significant differences between PD patients and controls.	0	
**Urine**	Jiménez-Jiménez et al., 1992 [98]	Flame atomic absorption spectrophotometry	Non-significant differences between PD patients and controls.	0	
	Forte et al., 2004 [28]	Inductively coupled plasma atomic emission spectrometry	Non-significant differences between PD patients and controls.	0	
	Bocca et al., 2006 [30]	Sector field inductively coupled plasma mass spectrometry	Significant decrease in PD patients.	2−	
	Fukushima et al., 2010, 2011, 2013 [50,51,52]	Atomic absorption spectrometry	Non-significant differences between PD patients and controls.	0	
	Ajsuvakova et al., 2020 [70]	Inductively coupled plasma atomic emission spectrometry	Non-significant differences between PD patients and controls.	0	
	Shan et al., 2025 [77]	Inductively coupled plasma atomic emission spectrometry	Significant increase in PD patients.	3+	
**Hair**	Bocca et al., 2006 [30]	Sector field inductively coupled plasma mass spectrometry	Significant increase in PD patients.	3+	
	Stefano et al., 2016 [79]	Inductively coupled plasma atomic emission spectrometry	Non-significant differences between PD patients and controls.	0	
	Ajsuvakova et al., 2020 [70]	Inductively coupled plasma atomic emission spectrometry	Non-significant differences between PD patients and controls.	0	

**Table 3:** **Comparison of techniques for measuring Cu, Zn, and Mn in biological tissues** **[117,118,119,120,121,122,123]**.

Technique	Principle	Sensitivity for Cu, Zn, Mn	Advantages	Limitations
Atomic Absorption Spectrophotometry (AAS)	Absorption of element-specific radiation by free atoms	Moderate	Reliable, relatively low cost	Single-element analysis
Flame Atomic Absorption Spectrophotometry (FAAS)	AAS with flame atomization	Moderate for Cu, Zn; low for Mn	Simple, rapid, inexpensive	Insufficient sensitivity for low Mn
Electrothermal Atomization/AAS (ETAAS)	Electrical heating atomization	High	High sensitivity, small sample volume	Matrix interferences
Graphite Furnace AAS (GFAAS)	AAS using graphite furnace	Very high	Excellent sensitivity	Low throughput, single-element
Inductively Coupled Plasma Spectroscopy (ICP-OES)	Optical emission from plasma-excited atoms	High	Multi-element capability	Higher cost than AAS
ICP–Sector Field Mass Spectrometry (ICP-SFMS)	Plasma ionization and high-resolution MS	Extremely high	Outstanding sensitivity/selectivity	Very high cost, complex
Monochromatic X-ray Fluorescence (XRF)	Characteristic X-ray emission	Moderate	Non-destructive	Limited sensitivity in soft tissues
Immunoturbidimetry	Antigen–antibody turbidity measurement	Indirect	Automated, clinical use	Does not measure metals directly

## Data Availability

The datasets used and/or analyzed during the current study are available from the corresponding author and/or José A. G. Agúndez on reasonable request.

## Supplementary Materials

The following supporting information can be downloaded at: https://www.mdpi.com/article/10.3390/cells15030288/s1, Supplementary Table S1. Results of studies addressing concentrations of copper, ceruloplasmin, zinc, and manganese in brain, CSF, serum/plasma, urine, and hair in patients with Parkinson’s disease compared to controls. Supplementary Table S2. PRISMA Checklist. Supplementary Table S3. MOOSE Checklist.
